# Efficacy and safety of belantamab-mafodotin in triple-refractory multiple myeloma patients: A multicentric real-life experience

**DOI:** 10.3389/fonc.2022.1026251

**Published:** 2022-11-15

**Authors:** Rossella Iula, Danilo De Novellis, Fabio Trastulli, Roberta Della Pepa, Raffaele Fontana, Angela Carobene, Maria Di Perna, Alessandro D’Ambrosio, Martina Romano, Aldo Leone, Laura De Fazio, Alfonso Fiumarella, Giuseppe Gaeta, Violetta Marafioti, Serafina Barbato, Salvatore Palmieri, Stefano Rocco, Bianca Serio, Catello Califano, Fabrizio Pane, Felicetto Ferrara, Valentina Giudice, Carmine Selleri, Lucio Catalano

**Affiliations:** ^1^ Hematology Unit, Department of Clinical Medicine and Surgery, University of Naples “Federico II”, Naples, Italy; ^2^ Hematology and Transplant Center, University Hospital “San Giovanni di Dio e Ruggi d’Aragona”, Salerno, Italy; ^3^ Department of Medicine, Surgery and Dentistry “Scuola Medica Salernitana”, University of Salerno, Baronissi, Italy; ^4^ Hematology and Transplant Program, AORN “A. Cardarelli” Hospital, Naples, Italy; ^5^ Onco-Hematology Unit, “A. Tortora” Hospital, Pagani, Italy

**Keywords:** anti-BCMA, multiple myeloma, refractory, multi-refractory, real life

## Abstract

Belantamab-mafodotin is an innovative and selective treatment for multi-refractory/relapsed multiple myeloma (MM) patients; however, available real-life experiences on efficacy and safety are limited. In this real-world multicentric retrospective study, we enrolled 28 MM patients treated in four Hematology units of Campania region, Italy, who received a median of six treatment lines prior to belantamab-mafodotin. The overall response rate (ORR) was 40% (complete remission, CR, 11%; very good partial remission, VGPR, 11%; and partial remission, PR, 18%), with a median progression-free survival (PFS) and overall survival (OS) of 3 and 8 months, respectively. One of the most frequent drug-related adverse events was keratopathy observed in nine (32%) patients, leading to therapy discontinuation in only three (11%) of them. Moreover, 22 out of 28 total patients who were treated with at least two administrations achieved an ORR of 50% (CR, 14%; VGPR, 14%; and PR, 22%) with a median PFS and OS of 5 and 11 months, respectively. In conclusion, our multicentric study confirmed efficacy and safety of belantamab-mafodotin in triple-refractory MM patients even in the real-life setting.

## Introduction

Multiple myeloma (MM), a clonal plasma cell (PC) disorder accounting for 10% of all hematological malignancies, is characterized by uncontrolled proliferation of neoplastic PCs with hyperproduction of monoclonal immunoglobulins (M-proteins) and end-organ damage (1). The median age at diagnosis is 69 years, and only less than 15% of patients are younger than 55 years (1). Therefore, in most cases, patients are not eligible for hematopoietic stem cell transplantation (HSCT), and alternative therapeutic strategies are considered (1). In recent years, the introduction of several novel classes of therapy has completely revolutionized MM outcomes with a significant improvement in overall survival (OS) (2). These new treatments include proteasome inhibitors (PIs), immunomodulatory agents (IMiDs), and anti-CD38 monoclonal antibodies (mAbs), variously combined and used in preclinical and clinical trials. However, MM is still considered an incurable cancer because of the inability to completely eradicate neoplastic PCs, as almost all patients ultimately relapse (1, 2). Moreover, several novel drugs are currently used as first-line treatments, and patients at relapse have already been exposed to the best available therapeutic options, making them defined as “triple-refractory” when previously treated with a PI plus an IMiD and an anti-CD38 mAb, or as “penta-refractory” when refractory to two PIs, two IMiDs, and one anti-CD38 mAb (3). MAMMOTH is the first study investigating clinical outcomes of MM patients refractory to an anti-CD38 mAb, and a significant decrease in OS is reported for “penta-refractory” subjects with a median survival of 5.6 months, an overall response rate (ORR) after at least one subsequent line of therapy of 31%, and a median progression-free survival (PFS) of 3.4 months (4, 5). Similar results for triple-refractory patients prospectively treated with selinexor, an XPO1 inhibitor, and dexamethasone have been reported in the STORM trial, with an ORR of 26% and median OS and PFS of 8.6 and 3.7 months, respectively (6).

Belantamab-mafodotin is a first-in-class antibody–drug conjugate consisting of an anti-B-cell maturation antigen (BCMA) mAb conjugated to a microtubule inhibitor monomethyl auristatin F and is approved for triple-refractory MM patients (7). BCMA, a member of the tumor necrosis factor receptor (TNFR) superfamily, plays a critical role in survival of bone marrow (BM) PCs, and its overexpression is associated with MM progression. Furthermore, BMCA is absent on naïve and memory B cells and is minimally expressed on hematopoietic stem cells while present on malignant MM PCs, promoting their survival (7–9). Belantamab-mafodotin is theoretically highly specific for neoplastic PCs by sparing normal B cells and other off-target cell types. In relapsed/refractory (R/R) MM, one-third of patients receiving belantamab-mafodotin at 2.5 mg/kg achieve an overall response, mostly a very good partial response (VGPR), at a median follow-up of 13 months, with a median PFS and OS of 2.8 and 13.7 months, respectively (10–12). Based on DREAMM-2 study results, belantamab-mafodotin received the approval as monotherapy at a dose of 2.5 mg/kg every 3 weeks in patients with R/R MM patients who have previously received four or more lines of therapy, including an anti-CD38 mAb, a PI, and an IMiD (7).

Belantamab-mafodotin toxicity profile is mostly characterized by monomethyl auristatin F class-related adverse events, with frequent keratopathy (all grades, 71%; grade III/IV, 44%) described as superficial and bilateral with microcyst-like lesions observed on slit lamp microscopy (13). Keratopathy-related symptoms are visual acuity (all grades, 53%; grade III/IV, 28%), blurred vision (all grades, 22%; grade III/IV, 4%), and dry eyes (all grades, 14%; grade III/IV, 1%); however, only 1% of patients discontinued the drug because of severe keratopathy (7, 14).

Data on the efficacy and safety of belantamab-mafodotin for treatment of R/R MM in real-life studies are few because of its recent approval. In this retrospective study, we reported a regional multicenter experience of the efficacy and safety of belantamab-mafodotin in R/R MM.

## Patients and methods

### Study cohort

Four Hematology Units of the Campania region in Italy participated in this study: University of Naples “Federico II,” University Hospital “San Giovanni di Dio e Ruggi d’Aragona” of Salerno, “Cardarelli” Hospital of Naples, and “A. Tortora” Hospital of Pagani, Salerno. Heavily pretreated (at least triple refractory) R/R MM patients were included in this study. Inclusion criteria were as follows: age ≥ 18 years old; diagnosis of MM according to 2014 International Myeloma Working Group (IMWG) criteria (15); R/R to at least one PI, one IMiD, and one anti-CD38 mAb; belantamab-mafodotin monotherapy outside clinical trials since June 2020. We first selected all patients who received at least one dose of belantamab-mafodotin (total population), and then only subjects who completed ≥2 cycles (evaluable population) were further studied. This study was conducted in accordance with the Declaration of Helsinki, the International Conference on Harmonization Good Clinical Practice guidelines (16), and protocols approved by our ethics committee Campania Sud, Brusciano, Naples, Italy (prot./SCCE n. 24988). All the patients provided written informed consent.

### Treatment

Belantamab-mafodotin was administered at the standard dose of 2.5 mg/kg by intravenous infusion (i.v.) every 3 weeks until disease progression or unacceptable toxicity prior intravenous premedication infusion with 20 mg of dexamethasone and 10 mg of chlorphenamine.

When patients started belantamab-mafodotin, keratopathy prophylaxis was performed with corticosteroids three times daily starting from day 1 to day 3 of the cycle, even though no clear benefits have been described in the DREAMM-2 study in preventing keratopathy (14). However, topical steroid eye drops have shown efficacy in corneal event mitigation for other drugs, such as high-dose cytarabine or mirvetuximab soravtansine, without significantly increasing adverse events (17, 18). Lubricant eye drops were prescribed afterward at least four times daily. In two centers, patients also received ice packs on eyes during drug infusion, based on previous studies suggesting clinical benefits of this procedure during belantamab-mafodotin or other anticancer agent administration (19, 20). Antibiotic, antiviral, and antifungal prophylaxis was conducted according to international guidelines (21).

Laboratory assessments were performed at baseline and before starting each cycle to evaluate disease status. All patients underwent eye examination by an ophthalmologist before the first belantamab-mafodotin cycle and every three administrations at University Hospital “San Giovanni di Dio e Ruggi d’Aragona” or as clinically indicated.

### Endpoints

Primary endpoint was ORR (ORR = partial remission [PR] + VGPR + complete remission [CR] + stringent CR [sCR]), based on modified IMWG response criteria, considering sCR and CR definition without performing BM aspirate (15, 22). Secondary end points were PFS, OS, clinical benefit rate (CBR, rate of patients with stable disease [SD]), response duration (DOR), time to response (TTR), time to best response (TTBR), and safety in total and evaluable cohorts. Safety was assessed as per the National Cancer Institute’s Common Terminology Criteria for Adverse Events version 4.0 (CTCAE v5.0).

### Statistical analysis

Data were collected in spreadsheets and were analyzed using R statistical software (v. 4.0.5; RStudio) and SPSS (v. 25; IBM). Differences between groups were investigated by chi-square, Fisher’s, Wilcoxon signed-rank, or unpaired two-tailed t-tests. Kaplan–Meier and log-rank tests were used for survival analysis, whereas Cox and logistic regression models were employed for investigation of the impact of independent variables on survival and outcomes. A P-value of <0.05 was considered statistically significant.

## Results

### Baseline characteristics

A total of 28 patients who intravenously received belantamab-mafodotin at 2.5 mg/kg every 3 weeks until disease progression or unacceptable toxicity were included in this retrospective real-world study (total cohort). Clinical characteristics are summarized in **Table 1**. The median age at first belantamab-mafodotin administration was 67.5 years (range, 51–83 years), and 16 patients were men (57%). IgG was the most common M-protein type (61%) with a median level of 1.2 g/dl (range, 0–7 g/dl). Eight patients (28%) had extramedullary disease before starting belantamab-mafodotin. Patients had a median glomerular filtration rate (GFR) of 71 ml/min (range, 8–125 ml/min), and 20 (71%) patients had renal impairment (GFR <90 ml/min) including four (14%) subjects with severe renal dysfunction (GFR <30 ml/min). Osteolytic lesions were more frequently occurring in 86% of patients (N = 24). At baseline, 12 (43%) patients were thrombocytopenic (11% grade I, N = 3; 18% grade II, N = 5; 3% grade III, N = 1; 11% grade IV, N = 3). Median time-to-belantamab-mafodotin treatment from diagnosis was 7 years (range, 1–19 years). Median number of previous therapies was 6 (range, 3–14), and all patients received at least one IMiD, one anti-CD38mAB (daratumumab in 100% of cases), and at least one PI (bortezomib in all cases, and carfilzomib administered as second PI in 86% of patients).

**Table 1:** Patients’ characteristics prior belantamab-mafodotin administration.

Characteristics	Total cohort N = 28	Evaluable cohort* N = 22
Median age, years (range)	67.5 (51-83)	66.5 (51-83)
Male/female, n (%)	16 (57)/12(43)	13 (59)/9(41)
M-protein type, n (%)		
IgG IgA Light chain Non-secretory	17 (61) 5 (18) 5 (18) 1 (3)	12 (56) 4 (18) 5 (22) 1 (4)
Light chain type, n (%)		
Kappa Lambda Non-secretory	15 (54) 12 (43) 1 (3)	12 (55) 11 (41) 1 (4)
Extramedullary disease	8 (28)	5 (23)
Prior auto-HSCT, n (%)	20 (71)	12 (67)
Prior treatment lines, median (range) Triple class refractory, n (%) Immunoregulatory drugs, n (%)	6 (3-14) 28 (100) 28 (100)	6 (3-14) 22 (100) 22 (100)
Anti-CD38 monoclonal antibodies		
- Daratumumab, n (%) - Isatuximab, n (%)	28(100) 0	22 (100) 0
Prior proteasome inhibitor treatment		
-Bortezomib, n (%) -Carfilzomib, n (%)	28 (100) 24 (86)	22 (100) 19 (86)
Median GFR, mL/min (range) GFR ≥90 ml/min, n (%) 60 ≤GFR <90 ml/min, n (%) 30 ≤GFR <60 ml/min, n (%) GFR <30 ml/min, n (%)	71 (8-125) 8 (29) 8 (29) 8 (29) 4 (13)	85 (8-125) 7 (32) 8 (36) 5 (23) 2 (9)
M-protein, g/dL, median (range) Osteolytic lesions, n (%)	1.2 (0-7) 24 (86)	1.1 (0-6.7) 20 (91)
Thrombocytopenia, n (%) Grades I–II, n (%) Grades III–IV, n (%)	12 (43) 8 (29) 4 (14)	10 (45) 7 (32) 3 (13)

Ig, immunoglobulin; auto-HSCT, autologous hematopoietic stem cell transplantation; GFR, glomerular filtration rate. *Evaluable cohort = patients treated with ≥2 belantamab-mafodotin cycles.

### Efficacy and clinical outcomes

**Figure 1:**
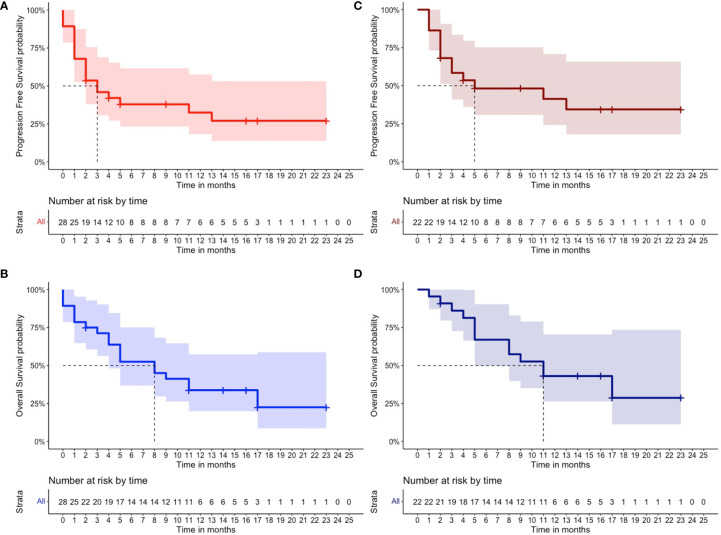
Clinical outcomes of patients receiving belantamab-mafodotin. **(A)** Progression-free survival and **(B)** overall survival of multi-refractory/relapsed multiple myeloma patients of total cohort (N = 28) who received belantamab-mafodotin, and **(C)** progression-free survival and **(D)** overall survival of multirefractory/relapsed multiple myeloma patients of the evaluable cohort (N = 22) who received at least two belantamab-mafodotin administrations are shown. The number of censored subjects at risk is also reported.

**Figure 2:**
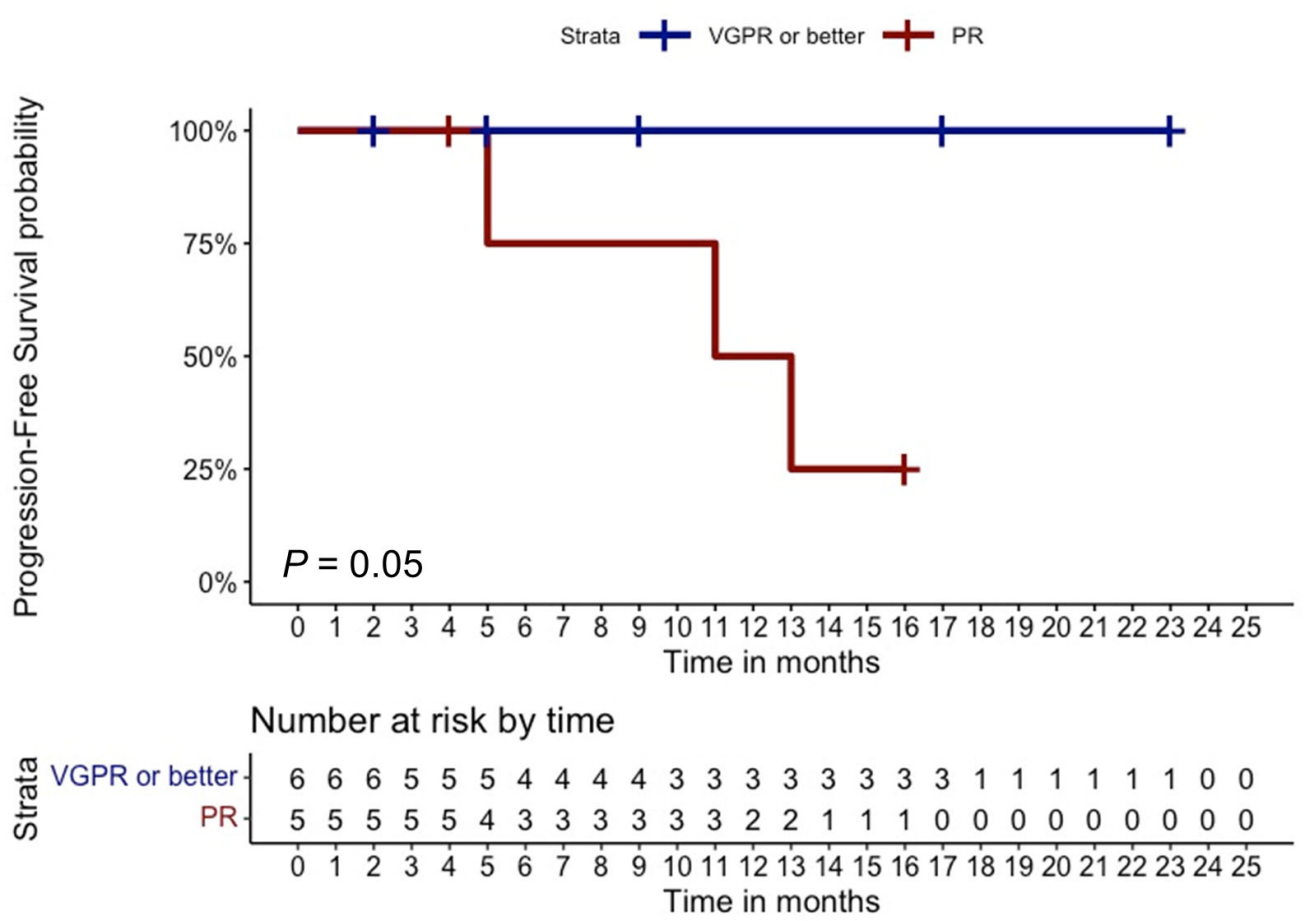
Clinical outcomes based on overall response. Progression-free survival of multi-refractory/relapsed multiple myeloma patients who achieved at least a very good partial response (VgPR) was compared to that of subjects who achieved a partial remission (PR). The number of censored subjects at risk is also reported. A P < 0.05 was considered statistically significant.

In the total cohort, 11 out of 28 patients (40%) achieved an overall response (11% CR, N = 3; 11% VGPR, N = 3; and 18% PR, N = 5), whereas three patients (11%) had SD with a CBR of 50% (N = 14) (**Table 2**). Notably, only one out of eight patients with extramedullary disease (12%) achieved a CR. At the time of writing, two out of the 11 patients with an overall response (18%) are still receiving belantamab-mafodotin. The remaining subjects stopped the treatment, because of consolidation with a second autologous hematopoietic stem cell transplantation (HSCT; N =2, 18%); severe ocular toxicity (N = 2, 18%); progressive disease (N = 3, 27%) after an initial response lasted for 3, 7, and 10 months, respectively; and treatment-unrelated deaths (N = 2; 18%) caused by SARS-CoV-2 infection or sepsis in subjects who have achieved a VGPR or CR, respectively. At a median follow-up of 6.5 months (range, 0–23 months), the median PFS was 3 months (range, 0–23) and the median OS was 8 months (range, 0–23) (**Figures 1A, B**), and the estimated 1-year PFS and OS were 32% and 34%, respectively. The median DOR was not reached (range, 2–23 months), the median TTR was 2 months (range, 1–5), and the median TTBR was 5 months (range, 2–14). Among patients with an overall response, achieving a VGPR or better was highly predictive of lasting responses, as the group of patients who achieved at least a VGPR did not reach a median PFS, whereas subjects who achieved only a PR had a median PFS of 11 months (hazard ratio, 83; P = 0.05). Notably, no disease progression occurred in the ≥VGPR cohort (**Figure 2**).

**Table 2:** Clinical outcomes and toxicities.

Characteristics	Total cohort N = 28	Evaluable cohort* N = 22
N. of cycles, median (range) Years from diagnosis to therapy, median (range) Steroid and lubricant eye drop premedication, n (%) Systemic steroid/antihistamine premedication, n (%)	3 (1-23) 7 (1-19) 28 (100) 28 (100)	4 (2-23) 7 (1-19) 22 (100) 22 (100)
Response rates		
ORR, n (%) CR, n (%) VGPR, n (%) PR, n (%) SD, n (%)	11 (40) 3 (11) 3 (11) 5 (18) 3 (11)	11 (50) 3 (14) 3 (14) 5 (22) 3 (14)
Median PFS, months (range) 1-year PFS Median OS, months (range) 1-year OS Median DOR, months (range) 1-year DOR	3 (0-23) 32% 8 (0-23) 34% - -	5 (0-23) 41% 11 (1-23) 43% N.r. (2-23) 74%
Keratopathy, n (%) Grades I–II Grades III–IV Drug-related discontinuation, n (%) Drug-related dose reduction, n (%) Drug-related administration delay, n (%)	9 (32) 6 (21) 3 (11) 3 (11) 1 (3) 3 (11)	
Ocular reported symptoms		
Blurred vision, n (%) Dry eyes, n (%) Severe BCVA reduction, n (%)	3 (11) 2 (7) 3 (11)	
Thrombocytopenia, n (%) Grades I–II Grades III–IV	13 (46) 9 (32) 4 (14)	

ORR, overall response rate; CR, complete remission; VGPR, very good partial remission; PR, partial remission; SD, stable disease; PFS, progression-free survival; OS, overall survival; DOR, response duration; BCVA, best corrected visual acuity. *Evaluable cohort = patients treated with ≥2 belantamab-mafodotin cycles.

For the subgroup of patients (N = 20; 71%) with renal impairment (GFR <90 ml/min), the ORR was 40% (N = 8, including four PR, three VGPR, and one CR). Patients were also divided based on renal impairment severity: mild (60 ≤GFR <90 ml/min; N = 8); moderate (30 ≤GFR <60 ml/min; N = 8); and severe (GFR <30 ml/min; N = 4). Subsequently, outcomes were compared within groups, showing no significant differences. In particular, ORRs were 50% (4/8; 2 PR and 2 VGPR) in patients with mild, 25% (2/8; 1 PR and 1 CR) moderate, and 50% (2/4; 1 PR and 1 VGPR) severe renal impairment, respectively.

Univariate analysis did not identify any independent variables associated with PFS, except for overall responses (**Table 3**). Nineteen patients (68%) died, six (21%) of them within first belantamab-mafodotin cycle likely due to an end-stage disease.

**Table 3:** Univariate analysis—progression-free survival (PFS).

Variable	HR	95% CI	*P* value
Sex (male)	0.6	0.2-1.8	0.38
Age	1	0.94-1.1	0.92
Heavy chain (IgG)	1.7	0.53-5.64	0.35
Light chain (k)	0.79	0.26-2.4	0.68
Years from diagnosis	0.92	0.77-1.1	0.38
Prior therapy lines	1.07	0.88-1.32	0.47
M-protein	1.1	0.81-1.51	0.51
OR (yes)	0.11	0.03-0.46	<0.05
VGPR or better (yes)	0.01	0-143	0.35
GFR ≥40 ml/min (yes)	0.91	0.25-3.35	0.89

HR, hazard ratio; CI, confidential interval; IgG, immunoglobulin G; OR, overall response; VGPR, very good partial remission; GFR, glomerular filtration rate.

(N = 28).

In the subgroup of MM patients (n = 22) treated with at least two belantamab-mafodotin cycles, the ORR, median PFS, and median OS were 50% (CR, 14%; VGPR, 14%; and PR, 22%), 5 months, and 11 months, respectively (**Figures 1C, D**). Clinical characteristics and outcomes of the evaluable cohort are displayed in **Table 2**.

### Safety

Belantamab-mafodotin was generally well tolerated in our population, and thrombocytopenia (N = 13; 46%) and keratopathy (N = 9; 32%) were the most frequent adverse reactions. Grade I–II thrombocytopenia occurred in nine (32%) and grade III–IV in four (14%) patients, respectively; however, no major bleedings were observed.

Three (11%) patients developed grade III keratopathy with associated severe changes in best-corrected visual acuity (BCVA), leading to permanent discontinuation treatment. In the remaining six cases, grade I–II ocular toxicity was managed with dose reduction (N = 1; 3%), administration delay (N = 3; 11%), or lubricant eye drops (N = 2; 7%). Blurred vision, dry eyes, and BCVA changes were the most reported symptoms, occurring in three (11%), two (7%), and three (11%) patients, respectively.

Corneal toxicity was transient and completely resolved after the end of belantamab-mafodotin treatment in all cases. No association with keratopathy occurrence was documented by univariate logistic regression analysis (**Table 4**).

**Table 4:** Univariate analysis—keratopathy (N = 28).

Variable	Odds ratio	95% CI	*P* value
Sex (male)	1.71	0.29-9.9	0.54
Age	0.99	0.9-1.1	0.89
Heavy chain (IgG)	0.22	0.03-1.37	0.10
Light chain (k)	1.07	0.19-5.91	0.93
Years from diagnosis	1.02	0.81-1.28	0.83
Prior therapy lines	0.95	0.67-1.35	0.75
M-protein	0.5	0.23-1.18	0.12
Number of belantamab-mafodotin cycles	1.08	0.93-1.26	0.28
OR (yes)	3.2	0.54-18.9	0.2
GFR ≥30 ml/min (yes)	0.36	0.04-2.81	0.33

CI, confidential interval; IgG, immunoglobulin G; OR, overall response; GFR, glomerular filtration rate.

Furthermore, no belantamab-mafodotin dose adjustment was required and no new safety concerns were observed in the special population with mild, moderate, or severe renal failure, and no infusion-related reactions were observed.

## Discussion

Belantamab-mafodotin, the first-in-class antibody–drug conjugate consisting of an anti-BCMA mAb conjugated to a microtubule inhibitor monomethyl auristatin F, is approved for triple-refractory MM patients with a starting dose of 2.5 mg/kg adjusted based on drug-related toxicity, especially keratopathy. Real-life data are important to correctly assess therapeutic effects of innovative drugs, such as belantamab-mafodotin since clinical trials enroll patients with selected conditions. Therefore, in our retrospective real-world multicentric study, we aimed to investigate clinical outcomes and safety of triple-refractory MM patients who have received belantamab-mafodotin. Our results confirmed the efficacy and safety of this novel anti-myeloma drug even in the real-world setting. Indeed, our real-life outcomes were similar to those reported in the DREAMM-2 trial (11). We reported an ORR and a CBR of 40% and 50%, respectively, similar to those observed in the 2.5-mg/kg cohort of the DREAMM-2 trial with an ORR of 31% (VGPR or better, 19%) and a CBR of 34%, respectively. In addition, the 1-year DoR, median PFS, and median OS in our cohort were 74%, 3 months, and 8 months, respectively, similar to those described in the DREAMM-2 study with a median DOR of 11 months, a median PFS of 2.9 months, and a median OS of 13.7 months (11, 12).

In a recent retrospective real-life experience, clinical outcomes from 36 heavily pretreated MM patients (median prior lines, 8) who have received belantamab-mafodotin monotherapy or in association with other anti-myeloma agents have been reported showing an ORR of 33% (mostly PR, 19%), a median DOR of 5 months, and a median PFS and OS of 2 and 6.5 months, respectively, at a median follow-up period of 6 months (23). Our slightly higher responses could be linked to the lower number of prior lines of treatments in our cohort (N = 6; range, 3–14) compared with the DREAMM-2 (N = 7), with the Mayo Clinic (N = 8) studies, and with our smaller sample size (N = 28) versus DREAMM-2 (N = 97) and Mayo Clinic (N = 36) experiences (11, 21). These promising clinical outcomes suggested that belantamab-mafodotin might be employed earlier in the treatment of MM to increase the response duration and prognosis, and achieving at least a VGPR could be a predictor of response maintenance; however, this finding needs to be confirmed in larger and prospective studies, by also comparing belantamab-mafodotin monotherapy or in combination with other anti-myeloma agents especially in patients with unsatisfactory partial responses. Moreover, outcomes were even more promising when considering the subgroup of patients who received at least two drug administrations, suggesting that anticipation of belantamab-mafodotin in earlier therapeutic lines could significantly improve clinical outcomes even in subjects with poor clinical conditions.

Renal impairment is frequently found in MM also at diagnosis, because of accumulation of monoclonal light chains in renal tissues, and disease progression, hypercalcemia, and nephrotoxic drugs are contributing factors of renal failure development that could require drug dosage adjustment or discontinuation (24). Our rate of patients with renal impairment was 71% (20/28), and this dysfunction was mild (60 ≤GFR <90 ml/min), moderate (30 ≤GFR <60 ml/min), and severe (GFR <30 ml/min) in 8/28 (29%), 8/28 (29%), and 4/28 (13%) subjects, respectively, slightly higher than that reported in the Mayo Clinic and DREAMM-2 trials, especially for severe renal failure. Notably, our patients with mild, moderate, or severe renal failure showed an ORR of 50%, 25%, or 50%, respectively, similar to that reported in DREAMM-2 and Mayo clinic trials (11, 23), even considering the special population of the DREAMM-2 trial with mild (N = 48) and moderate (N = 24) renal dysfunction. Indeed, also in this case, our ORR was similar to those already reported (50% vs. 33% and 25% vs. 33% for mild and moderate renal impairment, respectively) (25).

Moreover, one patient from our cohort was on dialysis while receiving belantamab-mafodotin for a total of 11 cycles achieving a VGPR, and he received the drug on days when he was not on dialysis without dose reduction or treatment delays. This patient did not experience keratopathy and died because of SARS-CoV-2 infection complications. Therefore, our results suggested that belantamab-mafodotin could be safely administered in MM patients with renal impairment without reducing clinical benefits, likely because the drug is mainly degraded and eliminated through internalization and intracellular proteolysis (26). Our findings are of great interest because patients with renal dysfunction are poorly represented in published clinical trials (e.g., only 2% of cases had renal impairment in the DREAMM-2 study) (11).

Interestingly, 19% of Mayo Clinic patients had been previously exposed to chimeric antigen T-lymphocytes (CAR-T), another innovative anti-BCMA therapy (23). In DREAMM-2 and in our trial, no patients underwent CAR-T therapy before BM administrations (11). In the future, the correct sequential use for all the novel anti-BMCA treatments should be defined because of absence of data about belantamab mafodotin after anti-BMCA CAR-T or vice versa, except for case report series (27).

Belantamab-mafodotin is safe with keratopathy and thrombocytopenia, the most frequent adverse drug-related reactions (11). No infusion-related reactions occurred. Of note, to increase the safety of drug administration in our patients, we deviated by our initiative from DREAMM-2 study indications, and a premedication with dexamethasone and chlorphenamine was used in the majority of patients, based on our experience accrued during infusion of other monoclonal antibodies. The absence of infusion-related events during initial administrations led to a steroid dose tapering, according to standard recommendations. Dexamethasone used as premedication could have had synergistic effects with other anticancer drugs; however, the effects of a single-dose administration in a setting of multi-treated and multi-refractory MM patients appear negligible.

Corticosteroid eye drops were prescribed as keratopathy prophylaxis at the beginning of our experience and then substituted with lubricant eye drops based on a DREAMM-2 ocular complication and management study, showing that corticosteroid eye drops do not reduce the incidence of keratopathy.

In our real-life experience, new-onset or worsening thrombocytopenia occurred in 46% (grades III–IV in 14%); however, no major bleedings occurred.

Keratopathy was also common (32% of cases). In one center (Salerno, N = 6), patients were evaluated by ophthalmologists every 3 months after starting belantamab-mafodotin, whereas in the other centers, visits were performed only in case of ocular clinical manifestations. This latter approach might better mirror real-life clinical management, as a close interdisciplinary assessment could be difficult to organize based on ophthalmologist availability and patients’ clinical conditions leading to access hospital the least possible. Therefore, the real rate of silent corneal damages might be underestimated. Grade III keratopathy occurred in three patients (14%) leading to treatment discontinuation also caused by occurrence of severe BCVA reduction. In the remaining cases, toxicity was managed with longer administration rates and dose reduction.

Our trial has several limitations. First, our study was a retrospective experience conducted on a small patient cohort, with a sample size lower than that from the DREAMM-2 trial but similar to that of the Mayo Clinic real-life experience (N = 36) (23). Second, cytogenetic alterations at diagnosis were missing, although the negative prognostic significance of these genetic alterations at onset might be less important in pluri-refractory MM. Third, there were no standardized procedures across different centers for keratopathy diagnosis and management. Fourth, the safety and efficacy of belantamab-mafodotin in patients with renal impairment were not systematically investigated, also because of the small number of patients, whereas results from the ongoing DREAMM-12 (NCT04398745) study are expected because the drug is evaluated also in patients with moderate, severe, or terminal renal impairment. Indeed, several trials are currently ongoing to evaluate belantamab-mafodotin efficacy and safety, such as the DREAMM-3 trial, comparing belantamab-mafodotin to pomalidomide/dexamethasone (NCT04162210), the phase I/II DREAMM-4 study (NCT03848845) investigating belantamab-mafodotin in combination with pembrolizumab or γ‐secretase inhibitor nirogacestat in the DREAMM-5 study (NCT04126200), or lenalidomide or bortezomib plus dexamethasone in the DREAMM-6 study (NCT03544281).

In conclusion, our real-life results were similar to those reported in the DREAMM-2 prospective trial and in the Mayo Clinic real-life experience showing the efficacy and safety of belantamab-mafodotin in R/R MM in a real-world setting and in longer follow-up.

## Data availability statement

The raw data supporting the conclusions of this article will be made available by the authors, without undue reservation.

## Ethics statement

The studies involving human participants were reviewed and approved by Ethics Committee “Campania Sud”, Brusciano, Naples, Italy (prot./SCCE n. 24988). The patients/participants provided their written informed consent to participate in this study.

## Author contributions

Conceptualization, FP, FF, LC, and CS; clinical data, RI, DN, FT, RD, RF, AC, MP, AD’A, MR, AL, LF, AF, GG, VM, SB, SP, SR, CC, BS, and VG; data curation, RI, DN, and VG; writing—original draft preparation, RI, DN, and VG; writing—review and editing, LC and CS. All authors have read and agreed to the published version of the manuscript.

## Acknowledgments

This research was supported by the Intramural Program of the Department of Medicine, Surgery and Dentistry, University of Salerno, Italy.

## Conflict of interest

The authors declare that the research was conducted in the absence of any commercial or financial relationships that could be construed as a potential conflict of interest.

## Publisher’s note

All claims expressed in this article are solely those of the authors and do not necessarily represent those of their affiliated organizations, or those of the publisher, the editors and the reviewers. Any product that may be evaluated in this article, or claim that may be made by its manufacturer, is not guaranteed or endorsed by the publisher.
